# Development and evaluation of a deep learning framework for pelvic and sacral tumor segmentation from multi-sequence MRI: a retrospective study

**DOI:** 10.1186/s40644-025-00850-8

**Published:** 2025-03-13

**Authors:** Ping Yin, Weidao Chen, Qianrui Fan, Ruize Yu, Xia Liu, Tao Liu, Dawei Wang, Nan Hong

**Affiliations:** 1 Department of Radiology, Peking University People’s Hospital, 11 Xizhimen Nandajie, Xicheng District, Beijing, 100044 P. R. China; 2 Institute of Research, InferVision, Ocean International Center, Chaoyang District, Beijing, 100025 China

**Keywords:** Pelvic and sacral tumors, Magnetic resonance imaging, Deep learning, 2.5D, Segmentation

## Abstract

**Background:**

Accurate segmentation of pelvic and sacral tumors (PSTs) in multi-sequence magnetic resonance imaging (MRI) is essential for effective treatment and surgical planning.

**Purpose:**

To develop a deep learning (DL) framework for efficient segmentation of PSTs from multi-sequence MRI.

**Materials and methods:**

This study included a total of 616 patients with pathologically confirmed PSTs between April 2011 to May 2022. We proposed a practical DL framework that integrates a 2.5D U-net and MobileNetV2 for automatic PST segmentation with a fast annotation strategy across multiple MRI sequences, including T1-weighted (T1-w), T2-weighted (T2-w), diffusion-weighted imaging (DWI), and contrast-enhanced T1-weighted (CET1-w). Two distinct models, the All-sequence segmentation model and the T2-fusion segmentation model, were developed. During the implementation of our DL models, all regions of interest (ROIs) in the training set were coarse labeled, and ROIs in the test set were fine labeled. Dice score and intersection over union (IoU) were used to evaluate model performance.

**Results:**

The 2.5D MobileNetV2 architecture demonstrated improved segmentation performance compared to 2D and 3D U-Net models, with a Dice score of 0.741 and an IoU of 0.615. The All-sequence model, which was trained using a fusion of four MRI sequences (T1-w, CET1-w, T2-w, and DWI), exhibited superior performance with Dice scores of 0.659 for T1-w, 0.763 for CET1-w, 0.819 for T2-w, and 0.723 for DWI as inputs. In contrast, the T2-fusion segmentation model, which used T2-w and CET1-w sequences as inputs, achieved a Dice score of 0.833 and an IoU value of 0.719.

**Conclusions:**

In this study, we developed a practical DL framework for PST segmentation via multi-sequence MRI, which reduces the dependence on data annotation. These models offer solutions for various clinical scenarios and have significant potential for wide-ranging applications.

**Supplementary Information:**

The online version contains supplementary material available at 10.1186/s40644-025-00850-8.

## Introduction

Pelvic and sacral tumors (PSTs) are rare and complex, posing significant challenges for diagnosis and treatment due to their anatomical location and diverse tissue characteristics [1, 2]. These tumors, whether primary or secondary, typically present as large soft tissue masses that can invade surrounding structures such as peripheral blood vessels, nerves, and pelvic organs [3–5]. Due to the rarity of PSTs and the complexity of the surrounding anatomical structures, it is often difficult for radiologists to discern sufficient visual features to accurately contour the tumors, which greatly hinders effective treatment planning and surgical intervention [6]. 

Magnetic resonance imaging (MRI) is a key modality for visualizing PSTs, with various scanning sequences providing detailed information about tissue structures. Commonly used MRI sequences for PSTs include T1-weighted (T1-w), T2-weighted (T2-w), diffusion weighted imaging (DWI), contrast-enhanced T1-weighted (CET1-w) [7, 8]. Multi-sequence MRI, along with fusion techniques, offers critical insights into the tumor’s stage, size, location, and relationship with adjacent structures. It is recommended by the Sarcoma European Latin-American Network (SELENT) guidelines for pre-surgical assessment of PSTs [9–11]. SELENT is a collaborative network focused on improving clinical outcomes in sarcoma care through the development of evidence-based Clinical Practice Guidelines (CPG) [11]. Despite the effectiveness of multi-sequence MRI, accurately segmenting PSTs remains a challenge for radiologists due to the complex and sometimes indistinguishable nature of PSTs and surrounding tissues [12]. This process is not only time-consuming but also requires significant clinical expertise, highlighting the need for automated, practical segmentation solutions.

Deep learning (DL) has proven particularly successful in medical image segmentation tasks, as it can extract high-level features for accurate semantic segmentation in an end-to-end manner [13]. Specifically, U-Net [14], renowned for its high performance and wide applicability, is the most commonly used DL model for segmentation tasks, and has inspired various important DL segmentation architectures, such as 3D U-Net [15], nnU-Net [16]. Lempart M et al. developed the Pelvic U-Net, which improved performance and reduced the average time required for the automated segmentation of organ-at-risk in the pelvic region using computed tomography (CT) images [17]. Liu X et al. proposed a 3D U-Net model for segmenting pelvic lymph nodes, achieving a Dice score of 0.85 and an AUC of 0.963 [18]. In addition, Hamabe A et al. developed an automated 3D U-net segmentation model for surgical simulation based on pelvic MRI, significantly reducing processing time [19]. However, these studies predominantly focus on segmenting anatomical structures or common lesions in the pelvic region, using U-Net-based models. Few studies address the automatic segmentation of PSTs, especially with flexibility for different MRI sequence combinations.

In this study, we proposed a practical DL framework for the automatic segmentation of PSTs based on multiple MRI sequences (T1-w, T2-w, DWI, and CET1-w) using a 2.5D U-Net and a fast annotation strategy. We aimed to provide a reliable and convenient alternative method using these available MRI sequences. To accommodate varying MRI sequence combinations, we developed a general all-sequence model capable of segmenting PSTs from any combination of these sequences, while also exploring the influence of different MRI sequence inputs on model performance. To balance accuracy and applicability, we further provided a concise T2-fusion model, which offers improved performance by fusing of T2-w and CET1-w sequences as input. Together, these two DL models were designed to better meet the demands of realistic clinical settings.

## Materials and methods

### Patients selection

**Fig. 1:**
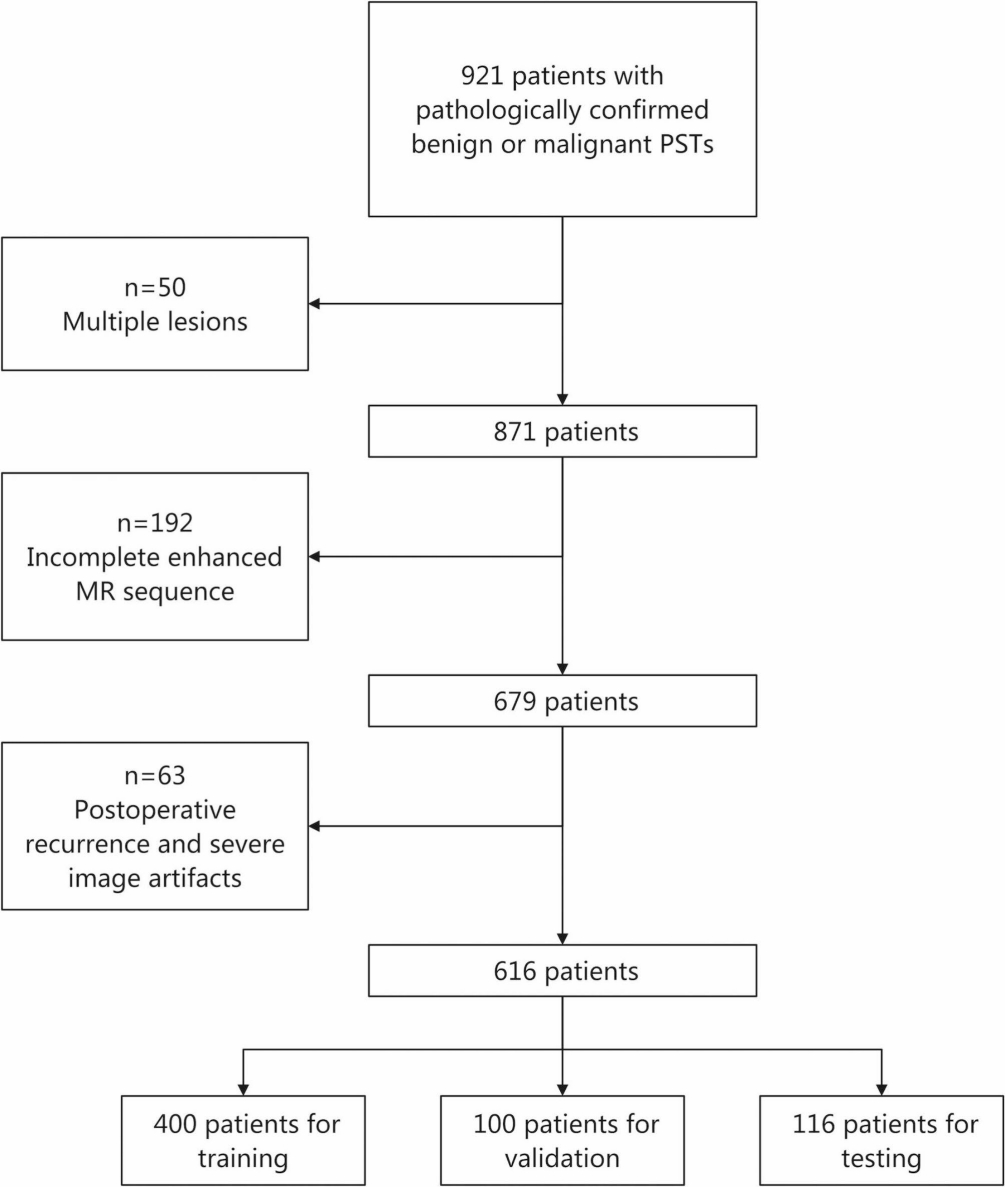
Flow chart of the selection procedure of included and excluded patients

This study retrospectively analyzed a cohort of 921 patients with pathologically confirmed benign or malignant PSTs who had undergone pelvic MRI at our hospital between April 2011 and May 2022. This study was approved by the institutional review board, and the reqirement for written informed consent was waived. The inclusion criteria were as follows: (1) Patients who had undergone MRI scans with all four sequences (T1-w, T2-w, DWI, and CET1-w) and whose images met diagnostic quality standards; (2) Patients with a single tumor lesion identified on MRI; (3) Pathological confirmation of benign or malignant PSTs. According to the WHO classification criteria, tumors classified as intermediate were grouped as benign in this study [20, 21]. Exclusion criteria included: (1) Patients with multiple tumor lesions on MRI (*n* = 50); (2) Patients with incomplete or missing enhanced MR sequences (*n* = 192); (3) Patients with postoperative recurrence or severe image artifacts that hindered analysis (*n* = 63). Ultimately, 616 patients with PSTs were included in the study and divided into three sets: training (*n* = 400), validation (*n* = 100) and internal test (*n* = 116). The dataset partitioning process ensured balanced representation of tumor types and imaging characteristics (e.g., T1-w, T2-w, DWI, and CET1-w) across the three sets. Specifically, stratified sampling was used to ensure that the distribution of benign and malignant tumors, as well as key MRI sequence characteristics, was proportionally represented in each set. This approach minimized potential biases in the model training process and enhanced generalizability. A summary of the dataset partitioning is provided in Table 1, and Fig. 1 illustrates the workflow of data enrollment.

**Table 1:** Clinical characteristic of patients

	Train^a^(*n* = 400)	Validation^b^(*n* = 100)	Test^c^(*n* = 116)	*p*-value
Age	41.83 ± 18.11	39.89 ± 16.78	43.04 ± 16.68	0.436
Size	10·00 ± 4.44	9.95 ± 4.61	9.93 ± 3.83	0.765
Sex				0.414
Male	219	56	56	
Female	181	44	60	
Location				0.544
Iliac crest	80	22	26	
Acetabulum	27	12	5	
Pubis/ischium	32	6	7	
Sacrum	206	45	63	
Multi-zone^d^	55	15	15	
Malignancy				0.799
Malignant	302	78	86	
Benign	98	22	30	

Note:

a, In the training set, the other tumor locations include 80 on iliac crest, 55 on multi-zone, 32 on pubis/ischium and 27 on acetabulum

b, In the validation set, the other tumor locations include 22 on iliac crest, 15 on multi-zone, 12 on acetabulum and 6 on pubis/ischium

c, In the test set, the other tumor locations include 26 on iliac crest, 15 on multi-zone, 7 on pubis/ischium and 5 on acetabulum

d, “multi-zone” is a tumor that involves more than one area simultaneously

### Imaging acquisition

Pelvic MRI acquisitions were performed on the Signa HDxt 3.0T (GE Healthcare), Signa EXCITE 1.5T (GE Healthcare), and Discovery 750 3.0T (GE Healthcare, Waukesha, WI, USA). The detailed acquisition parameters were as follows: (1) axial T1-w with volume acceleration-flexible (LAVA-Flex) or T1-w Fast Spin Echo with Fat Suppression (FSE fs), repetition time (TR) = 3.8 ~ 700 ms, echo time (TE) = 1.7 ~ 7.8 ms, slice thickness = 4 ~ 7 mm, matrix = 288 × 224 ~ 320 × 224, and field of view (FOV) = 38 cm × 38 cm ~ 42 cm × 42 cm; (2) T2-w, TR = 2300 ~ 5119 ms, TE = 84.1 ~ 102.5 ms, slice thickness = 6 ~ 7 mm, matrix = 288 × 224 ~ 320 × 224, and FOV = 38 cm × 38 cm ~ 44 cm × 44 cm; (3) DWI, b value = 1000, TR = 4800 ~ 5000 ms, TE = 59.2 ~ 60 ms, slice thickness = 6 ~ 7 mm, matrix = 128 × 128 ~ 160 × 160, and FOV = 36 cm × 36 cm ~ 44 cm × 44 cm. (4) Axial CET1-w, LAVA-Flex was performed by intravenous injection of 0.2 mL/kg contrast medium (gadopentetate dimeglumine injection, Guangzhou Consun Pharma) with a high-pressure syringe or manual push, TR = 3.8 ~ 700 ms, TE = 1.7 ~ 7.8 ms, slice thickness = 4 ~ 7 mm, matrix = 288 × 224 ~ 320 × 224, and FOV = 38 cm × 38 cm ~ 42 cm × 42 cm.

### Manual annotation

Manual annotations of the retrospective dataset were performed using segmentation software (ITK-SNAP, version 3.6.0, www.itksnap.org) [22]. All regions of interest (ROIs) in the training set were coarsely labeled (5 layers above and below the largest level of the lesion), while ROIs in the test set were finely labeled (all layers of the lesion). A musculoskeletal radiologist with 7 years of experience (PY) delineated the tumor boundaries on each slice. A senior musculoskeletal radiologist with 15 years of experience (XL) validated the segmentation results.

### Proposed deep learning-based framework

**Fig. 2:**
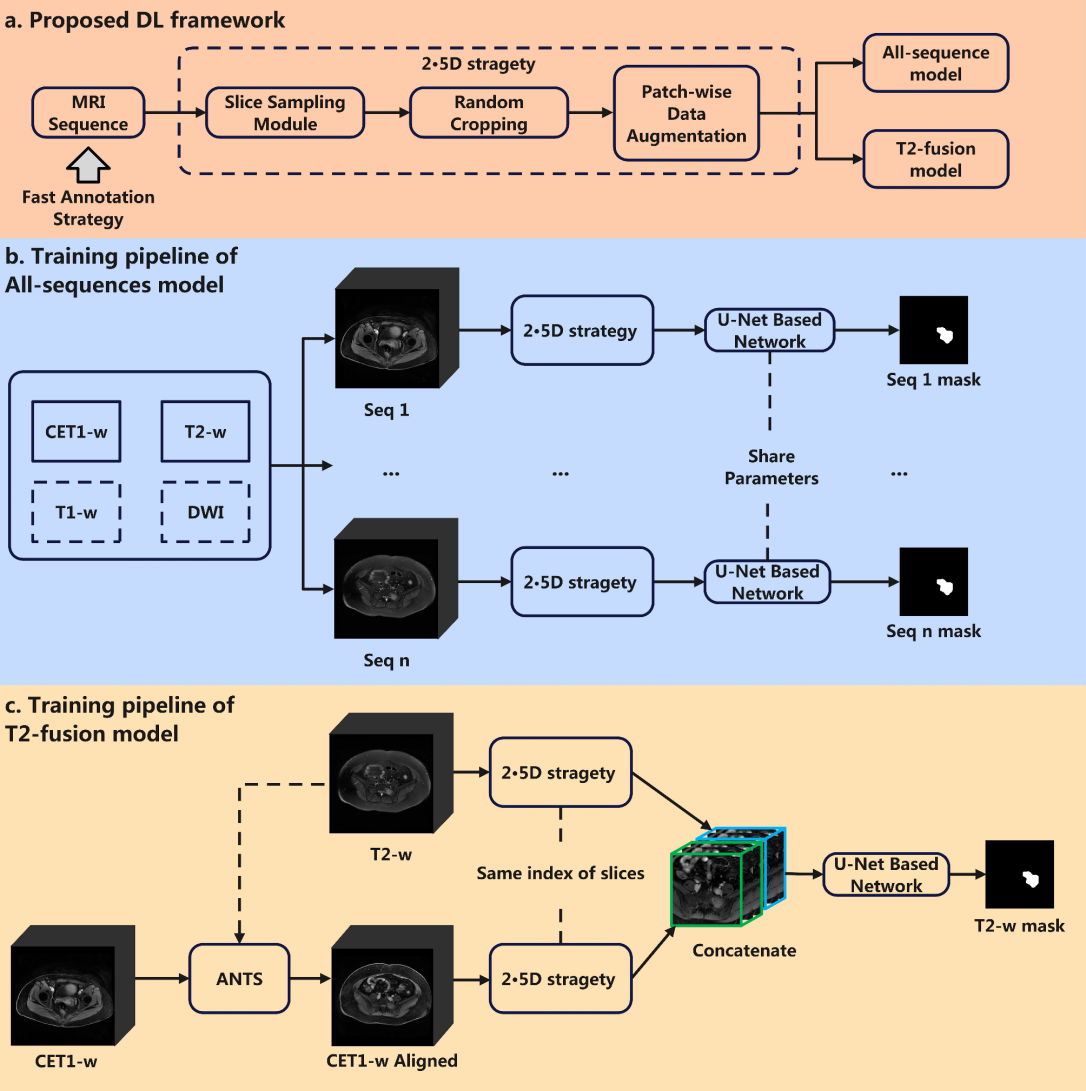
Structure of the 2.5D U-Net based deep learning models

To enhance the feasibility of model development and improve the applicability of DL model, we developed a DL framework with a fast annotation strategy for PSTs segmentation, as shown in Fig. 2. This framwork primarily consists of six key procedures. The fast annotation strategy involves manually delineating the tumor regions on a subset of the MRI images, which were selected based on the physician’s judgment, thus reducing labeling time. To ensure optimal performance of the DL models, we initially investigated three common DL modeling methods for PST segmentation: 3D [15], 2D [23], and 2.5D [24] strategies. After comparing their performance, we selected the 2.5D strategy as the optimal approach for constructing the DL framework. The 2.5D strategy integrates three modules—Slice Sampling, Random Cropping, and Patch-wise Data Augmentation—which effectively utilize spatial information from a limited number of labeled MRI images, while balancing computational efficiency and spatial context. While 3D models capture full spatial context, they require large datasets, which posed a challenge in this study. Conversely, 2D models are computationally efficient but fail to capture the 3D spatial relationships necessary for accurate segmentation of tumors with complex shapes. The 2.5D strategy addresses these challenges by processing multiple 2D slices from the tumor and surrounding regions, thereby capturing both local and broader spatial features. This approach maximizes the use of limited annotated data and reduces computational demands compared to 3D models, making it the most suitable choice for our dataset. As a result, the 2.5D model provides an effective balance between segmentation accuracy and efficiency.

Next, two U-Net based DL models were developed: the All-sequence segmentation model and the T2-fusion segmentation model. The All-sequence segmentation model is capable of performing PST segmentation with any combination of MRI sequences, improving its applicability in real-world clinical scenarios. Furthermore, we evaluated the influence of different MRI sequence combinations as inputs for the DL model. Notably, since CET1-w and T2-w have great clinical value, all combinations of MRI sequences included at least one of these two sequences. To further investigate these sequences, we employed an input-level fusion strategy to combine CET1-w and T2-w, resulting in the development of the T2-fusion model, which exhibited improved segmentation performance compared to the All-sequence model.

### All-sequence model and T2-fusion model

For the All-sequence model, all four MRI sequences were used during model training and fed into the model separately. During training, we first normalized the input sequences. The Slice Sampling Module was then applied to randomly select 12 slices from a patient’s four MRI sequences. The selection was random, and some of the slices contained annotations (representing lesions), while others did not. For each of the 12 randomly selected slices, the module also included the slice immediately above and below it, forming a 3-slice “image bag” for each selection. These “image bags” consisted of 12 sets, with each set containing 3 slices, forming 12 three-channel 2.5D images that were then input into the model for training. To address the coarse segmentation issue, we used a Random Cropping Module to crop the images to a uniform size of 12 × 3 × 224 × 224. We chose the 224 × 224 image size for two main reasons. First, our calculations showed that this size effectively covers the lesion area while reducing memory usage. Second, 224 × 224 was widely used in DL applications, offering an optimal balance between capturing detailed features and minimizing computational load, making it a practical choice for both performance and efficiency. The cropped images were saved in the same format, preserving tumor features and intensity values, ensuring no information was lost during the process. Data augmentation techniques—including Horizontal Flip, Vertical Flip, Shift, Scale, and Rotation—were applied to the cropped images to reduce overfitting (Patch-wise Data Augmentation Module). The final input shape of the All-sequence model was 12 × 3 × 224 × 224. During testing, the data preprocessing steps were skipped, and each slice of MRI sequences was fed into the segmentation model, along with the slices above and below it, separately.

For the training process of the fusion model, we first aligned CET1-w sequence to the spatial coordinates of T2-w by applying advanced normalization tools (ANTS) method to ensure the sequences had the same dimensions [25]. After aligning the two sequences, we applied the same 2.5D strategy modules used in the All-sequence model to both CET1-w and T2-w sequences. All operations were identical across the two sequences to ensure that the transformed images represented the same region. The two image patches were then concatenated channel-wise in an early fusion manner [26]. The final input shape of the fusion model was 12 × 6 × 224 × 224.

### Implementation details

U-Net has demonstrated impressive performance in medical image segmentation tasks [14], while MobileNetV2 is renowned for its rapid training speed and robust feature extraction capabilities [27]. Therefore, we adopted a U-Net architecture with an ImageNet-pretrained MobileNetV2 encoder as our segmentation model. The complete pipeline of our proposed DL framework and the training procedures for both segmentation models are shown in Fig. 2. The structure of the U-Net-based network is shown in Supplementary Fig. 1. Our segmentation model contains 6.63 million parameters and has a computational complexity of 2.6 giga lloating point operations per second (GFLOPs).

We implemented our models using Python 3.7 and PyTorch 1.11.0 on two Nvidia RTX3090 graphics cards. The models were trained for 300 epochs, with a batch size of 12. We used the AdamW optimizer with an initial learning rate of 0.0005 and employed Dice loss to optimize the models. The operating system was Ubuntu 20.04 with CUDA version 11.3. The code of this study is available at: https://github.com/TXVision/PSTs_segmentation_multiple_MRI_sequences.

### Model evaluation and statistical analysis

To evaluate the segmentation performance of the model, we used two metrics: the Dice score and Intersection over union (IoU). The Dice score is calculated as 2×Area of overlap/Total number of pixels in both images, while IoU is computed as the area of overlap divided by the area of union of both images. Both Dice and IoU scores range from 0 to 1, with 0 indicating no overlap and 1 indicating perfect overlap. Statistical analysis was performed using R software (R Core Team, Vienna, Austria, version 3.4.3). One-way ANOVA was employed to compare continuous variables, while the chi-squared test was used for categorical variables between groups. All statistical tests were two-sided, with a p-value less than 0.05 considered statistically significant.

## Result

### Characteristic of patients

A total of 616 patients (331 males, 285 females; mean age of 41.74 ± 17.63 years) were included in this study and divided into training set, validation set, internal test set (Table 1). The training set included 400 patients (219 males, 181 females; mean age of 41.83 ± 18.11 years), with a mean tumor size of 10.00 ± 4.44 cm. This set comprised 302 patients with malignant tumors and 98 patients with benign tumors. The most common tumor location was the sacrum, with 206 patients having tumors in this area. The validation set consisted of 100 patients (56 males, 44 females; mean age of 39.89 ± 16.78 years), with a mean tumor size of 9.95 ± 4.6 cm. This group included 78 malignant tumors and 22 benign tumors, with the sacrum being the most frequent tumor location, affecting 45 patients. The test set included 116 patients (56 males, 60 females; mean age of 43.04 ± 16.68 years), with a mean tumor size of 9.93 ± 3.83 cm. Of these, 86 patients had malignant tumors and 30 had benign tumors. As with the other sets, the sacrum was the most common tumor location, affecting 63 patients. There were no significant differences in age, sex, tumor size, tumor location, or malignancy level among the training, validation, and testing sets for our segmentation model (all *p* > 0.05) (See Table 1 for details).

### Comparison of 2D, 3D and 2.5D segmentation

The 2.5D MobilenetV2 + Unet model achieved the highest average Dice score of 0.741 and IoU value of 0.615. In contrast, the 3D nnUnet model had an average Dice score of 0.639 and an IoU value of 0.509, while the 2D MobilenetV2 + Unet model had an average Dice score of 0.693 and an IoU value of 0.560. These results suggest that the proposed 2.5D segmentation model outperforms both 2D and 3D-based approaches. (Table 2).

**Table 2:** Performance of different input dimensions

Input dimension	Model	Dice Score^a^	IoU^b^
3D	nnUnet	0.639	0.509
2D	MobilenetV2 + Unet	0.693	0.560
2.5D	MobilenetV2 + Unet	0.741	0.615

Note:

a, Dice score is 2×the area of overlap divided by the total number of pixels in both images

b, IoU is the area of overlap divided by the area of union of both images

### All-sequence model performance

To evaluate the performance of the 2.5D MobilenetV2 + Unet model, we explored various input-level fusion strategies.

Single Sequences: When training with individual MRI sequences, the Dice scores for each sequence were as follows: 0.589 for T1-w, 0.804 for T2-w, 0.804 for DWI, and 0.714 for CET1-w. Among these, both the T2-w and DWI sequences achieved the highest Dice score of 0.804, indicating superior performance in segmentation.

Fusion of Two Sequences: When combining two MRI sequences for training input, the T2-w & CET1-w fusion model achieved the best performance, with Dice scores of 0.804 for T2-w and 0.810 for CET1-w.

Fusion of Three Sequences: When combining three MRI sequences for training input, the CET1-w, T2-w, & DWI fusion model achieved the best performance, with Dice scores of 0.801 for CET1-w, 0.806 for T2-w and 0.701 for DWI.

All-sequence Model: The All-sequence model, which used T1-w, CET1-w, T2-w, and DWI as training inputs, resulted in Dice scores of 0.659 for T1-w, 0.763 for CET1-w, 0.819 for T2-w and 0.723 for DWI.

**Fig. 3:**
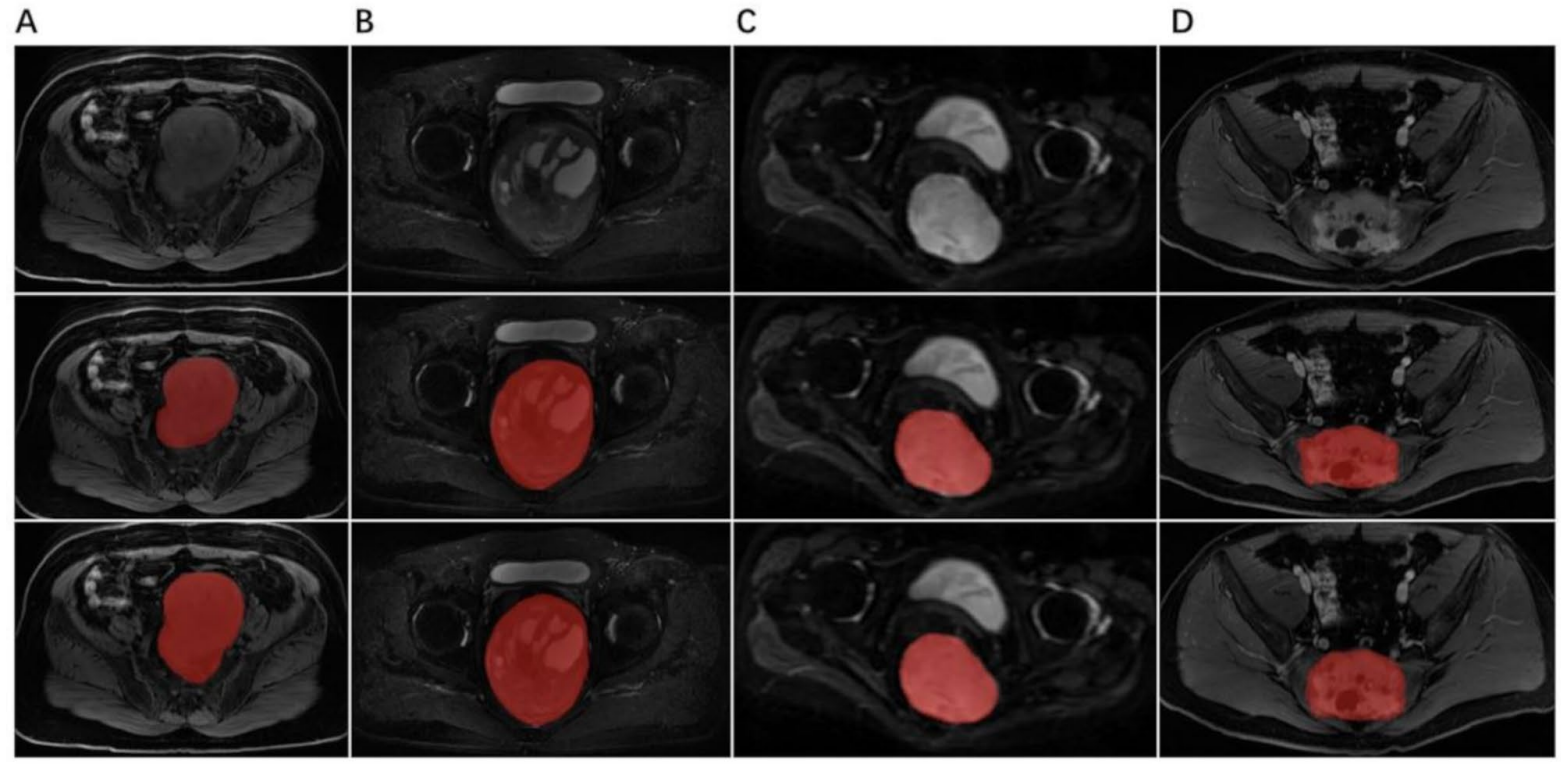
Comparison of Segmentation Results Across Different Cases in the All-sequence Model. (**A**) T1-w sequence, showing the image of a 62-year-old female patient diagnosed with neurofibroma. (**B**) T2-w sequence, showing the image of a 36-year-old male patient diagnosed with schwannoma. (**C**) DWI sequence, showing the image of a 45-year-old female patient diagnosed with schwannoma. (**D**) CET1-w sequence, showing the image of a 30-year-old male patient diagnosed with giant cell tumor of bone. The first row shows the original images, the second row presents the manual segmentation results, and the third row shows the model’s predicted segmentation results. The segmentation performance for T2-w is superior to that of the other sequences

Among all these results, we found that T1-w, T2-w and DWI inputs achieved the best performance in the All-sequence model. And the CET1-w input achieved the best performance in the T2-w & CET1-w model. These finding showed that the performance of our All-sequence model achieved the best segmentation ability. Segmentation results of different sequences are shown in Table 3. Figure 3 illustrates a comparison of segmentation results across different cases in the All-sequence model.

**Table 3:** Dice score^a^ of different input fusions for 2.5D MobilenetV2 + Unet

Input	T1-w	CET1-w	T2-w	DWI
T1-w	0.589			
CET1-w		0.804		
T2-w			0.804	
DWI				0.714
T1-w & CET1-w	0.645	0.767		
T2-w & CET1-w		0.810	0.804	
DWI & CET1-w		0.805		0.689
T1-w & T2-w	0.655		0.801	
DWI & T2-w			0.814	0.723
T1-w & T2-w & DWI	0.635		0.792	0.694
T1-w & T2-w & CET1-w	0.630	0.752	0.786	
T1-w & DWI & CET1-w	0.598	0.716		0.710
CET1-w & T2-w & DWI		0.801	0.806	0.701
All-sequence model^b^	0.659	0.763	0.819	0.723

Note:

a, Dice score is 2×the area of overlap divided by the total number of pixels in both images

b, All-sequence model represents the fusions of T1-w & CET1-w & T2-w & DWI

### T2-fusion model performance

The T2-fusion model was constructed using both T2-w and CET1-w sequences to generate the segmentation mask for T2-w, achieving a Dice score of 0.833 and an IoU value of 0.719. In contrast, when the T2-w sequence was used as input for the All-sequence model, it achieved a Dice score of 0.819 and an IoU value of 0.707. As shown in Table 4, this results demonstrated that the segmentation performance of the T2-fusion model is superior to that of the All-sequence model.

**Table 4:** Dice and IoU of all-sequence and T2-fusion model on T2

Model performance	Dice score^c^	IoU^d^
All-sequence model^a^	0.819	0.707
T2-fusion model^b^	0.833	0.719

Note:

a, All-sequence model represents the fusions of T1-w & CET1-w & T2-w & DWI

b, T2-fusion model was constructed by the T2-w & CET1-w preprocessed image patches and took the T2-w sequence as input

c, Dice score is 2×the area of overlap divided by the total number of pixels in both images

d, IoU is the area of overlap divided by the area of union of both images

## Discussion

The diagnosis and surgical planning of PSTs can be challenging due to their complex anatomical structures. Segmentation is an essential image processing step for identifying target areas, but it is often time-consuming. In this study, we present two models: the All-sequence model and the fusion model, both applied to four different MRI sequences and their combinations. The All-sequence mode achieved satisfactory segmentation performance with Dice score of 0.659 for T1-w, 0.763 for CET1-w, 0.819 for T2-w and 0.723 for DWI. The T2-fusion model showed even higher performance, achieving a Dice score of 0.833 for T2-w. These models have great potential for use in various clinical scenarios and could significantly enhance the automatic segmentation of PSTs.

Our study included 616 patients with PSTs and collected T1-w, CET1-w, T2-w, and DWI images from them. Since PSTs are rare, previous studies either had fewer patients or MRI sequences [18, 19, 21]. During manual segmentation of PSTs, the physicians labeled each slice precisely, but not all slices containing ROIs were annotated. This labeling method significantly reduced the labeling time required for physicians. We designed a 2.5D data preprocessing technique to form a DL-based framework for handling this type of coarse annotation. The use of 2.5D training techniques in the MobilenetV2 + Unet model has been shown to be an effective method for segmenting volumetric spatial information in MRI images and achieved the best performance compared with 2D and 3D inputs. 2D images struggle to present volumetric spatial information [28], while 3D CNNs require increased parameters, higher computation cost, and precise labels, which may not be suitable for PST images [28, 29]. This approach, combined with a carefully designed data preprocessing method, is particularly well-suited for segmenting rough and incontiguous labels, and has been verified to be effective in several volumetric segmentation studies. For instance, Yoganathan et al. showed that 2.5D DL models could outperform 2D models in automatically segmenting targets and organs-at-risk in MRI images of cervical cancer [30]. Shapey et al. proposed a novel DL framework that utilized 2.5D CNNs to automatically segment and calculate the volume of vestibular schwannomas tumors in MRI images, demonstrating that this approach could achieve performance equivalent to that of human annotators [31]. Similarly, Robert et al. used 2.5D U-Nets to segment bone templates in knee MRIs and achieved a Dice score of 98%, which was favorable in comparison to other algorithms [32]. Xie et al. developed the TransResSEUnet2.5D network, which utilized 2.5D techniques to segment the Gross Target Volume of lung cancer in CT images, outperforming five other segmentation models [33]. These studies highlight the clinical potential of 2.5D segmentation methods for accurately identifying bone and joint cancers. While 2.5D segmentation methods have been explored in other medical imaging tasks, our study uniquely addresses the segmentation of PSTs using multi-sequence MRI, a subject that has not been extensively studied. Additionally, our approach incorporates a novel multi-sequence fusion strategy that optimizes the use of T2-w and CET1-w sequences, specifically tailored to the characteristics of PSTs, and allows for effective segmentation even with coarse annotations.

We tried fourteen input strategies to train our 2.5D segmentation method, including T1-w, T2-w, DWI and CET1-w sequences individually, as well as various combinations of these sequences. All these combinations must include at least one of CET1-w or T2-w sequences, as these two sequences are crucial for diagnosing bone tumors in real clinical application. T2-w images are commonly used to locate tumors due to their ability to detect tumor density and provide the best depiction of disease [8], while CET1-w images may reflect intratumoral architecture and heterogeneity, such as tumor angiogenesis [7]. The results revealed that the All-sequence model achieved the satisfying segmentation ability on T1-w, T2-w, CET1-w and DWI as input, outperforming other models that used fewer sequences. This suggests that incorporating multiple sequences of MRI images can provide more radiographic information for segmentation tasks. This finding is supported by previous research in different tissues. For instance, Huang et al. developed a multi-sequence MRI based network for segmenting organs-at-risk in the pelvic cavity and found that fusing three sequences (T1-T1DIXONC-T2) resulted in better performance compared to fusing two sequences (T1-T2 and T1-T1DIXONC) [34]. To improve brain tumor detection, Amin et al. proposed a fusion method that combines information from four different MRI sequences (CET1-W, T1, FLAIR, and T2), and found that this approach led to better results than using individual sequences alone [35]. Similarly, Ye et al. utilized CNNs to develop a model for segmenting nasopharyngeal carcinoma and found that utilizing dual-sequence MRI (T1W and utilized T2W) resulted in more accurate segmentation than using a single sequence [36]. These results suggest that incorporating multiple sequences of MRI may provide more clinically useful information.

From Table 3, we can see that the combination of CET1 and T2-w achieved satisfactory segmentation results. As discussed earlier, T2-w and CET1-w sequences are particularly important for delineating PST borders. Therefore, we also constructed the T2-fusion model by using both T2-w and CET1-w images as input. This model achieved better segmentation results on T2-w images compared to the All-sequence model. For diagnostic purposes, T2-w and CET1-w images are often combined, as this combination allows for clearer delineation of PST borders. The fusion of T2-w and CET1-w images provides more radiomic information, and this approach has been widely demonstrated in previous literature [7, 37–39]. 

In general, the All-sequence model and the T2-fusion model each have their own advantages. The All-sequence model can generate accurate segmentation results when trained with any one of T1-w, DWI, CET1-w, or T2-w images as input, making it more convenient to use for clinical diagnosis in a real-world setting. In contrast, the T2-fusion model requires both T2-w and CET1-w images as input and can achieve higher segmentation performance on T2-w images. Thus, if both T2-w and CET1-w sequences are available, the T2-fusion model is a better option compared to the All-sequence model. The number of parameters and computational complexity of the U-Net-based network are relatively small compared to existing segmentation networks, making it more applicable in real-world implementations [40]. Besides of these two models, the DL-based framework we proposed for coarse labels could also be applied to other similar scenarios. In the All-sequence model, we found that CET1-w inputs performed relatively poorly, especially when combined with other sequences. This may be because CET1-w provides less effective tumor detection information compared to T2-w. While multi-sequence fusion generally adds more data, it doesn’t always improve performance, particularly when CET1-w’s contribution is limited. Future work will focus on optimizing the fusion strategy to enhance model performance.

Despite the promising performance of our models, several potential limitations should be noted. Firstly, all MRI scans were obtained from one institution, which may limit generalizability. Further research should include data from multiple centers to improve the robustness of the model. Secondly, our sample excluded the patients with incomplete or poor-quality images, which may introduce selection bias and limit the applicability. Thirdly, only single PSTs were included while multiple PSTs are common in clinical practice. In future work, we will extend the model to handle multiple-tumor cases and evaluate its performance using the existing validation and test sets. This will help assess how well the model performs in more clinically relevant scenarios with multiple tumors. Finally, while the models have demonstrated good performance in this study, further research is needed to assess their clinical utility in actual practice.

In conclusion, we have developed two highly-performing models for segmenting PSTs in multi-sequence MRI using a 2.5D U-Net approach. The All-sequence model is suitable for single-sequence images, while the T2-fusion model is optimized for T2-w image. These models have the potential to improve clinical efficiency and alleviate the workload of doctors. Our study is the first to apply 2.5D U-Net to PST segmentation based on the multi-sequence MRI images, and our results demonstrate the great potential for diagnosis, surgical planning, and treatment.

## Electronic supplementary material

Below is the link to the electronic supplementary material.

Supplementary Material 1

## Data Availability

No datasets were generated or analysed during the current study.

## Abbreviations

PSTs: Pelvic and sacral tumors
CT: Computed tomography
MRI: Magnetic resonance imaging
AI: Artificial intelligence
DL: Deep learning
DWI: Diffusion weighted imaging
CET1-w: Contrast-enhanced T1-weighted
ROIs: Regions of interest
IoU: Intersection over union
