# Supplementary material for: Development and evaluation of a deep learning framework for pelvic and sacral tumor segmentation from multi-sequence MRI: a retrospective study
Source: Cancer Imaging. 2025 Mar 13;25:34. doi: 10.1186/s40644-025-00850-8 (PMC11907785; doi:10.1186/s40644-025-00850-8)
Supplement: Supplementary file 1 — Supplementary Material 1 [file 40644_2025_850_MOESM1_ESM.docx]

**Supplementary appendix**

**Supplementary Figure 1**


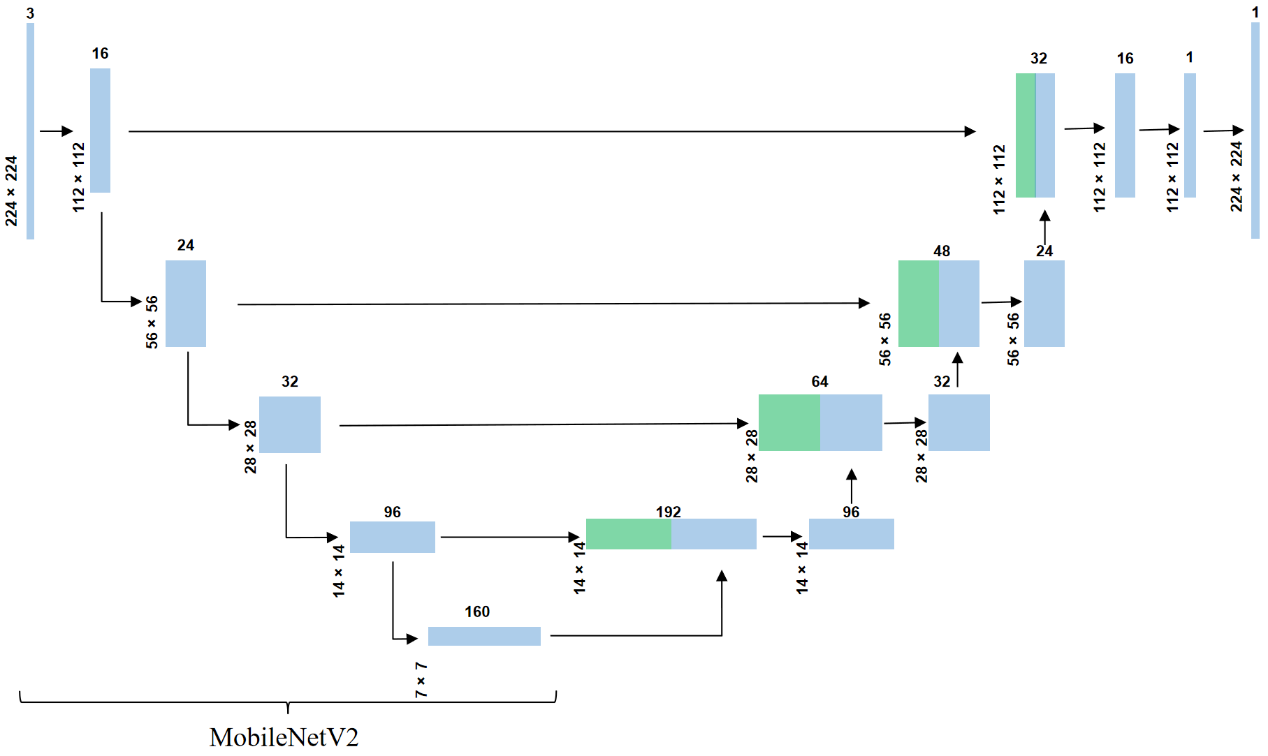


**Supplementary Figure 1. Illustration of U-Net-based network.**
